# Insertion of a myc-tag within α-dystroglycan domains improves its biochemical and microscopic detection

**DOI:** 10.1186/1471-2091-13-14

**Published:** 2012-07-26

**Authors:** Simona Morlacchi, Francesca Sciandra, Maria Giulia Bigotti, Manuela Bozzi, Wolfgang Hübner, Antonio Galtieri, Bruno Giardina, Andrea Brancaccio

**Affiliations:** 1 Istituto di Chimica del Riconoscimento Molecolare (CNR) c/o Istituto di Biochimica e Biochimica Clinica, Università Cattolica del Sacro Cuore, Largo F. Vito 1, 00168, Roma, Italy; 2 Dipartimento di Biologia Animale ed Ecologia Marina, Università degli Studi di Messina, Piazza Pugliatti 1, 98122, Messina, Italy; 3 Istituto di Biochimica e Biochimica Clinica, Università Cattolica del Sacro Cuore, Largo F. Vito 1, 00168, Roma, Italy; 4 EMBL, Structural and Computational Biology Unit, Meyerhofstraße 1, 69117, Heidelberg, Germany

## Abstract

**Background:**

Epitope tags and fluorescent fusion proteins have become indispensable molecular tools for studies in the fields of biochemistry and cell biology. The knowledge collected on the subdomain organization of the two subunits of the adhesion complex dystroglycan (DG) enabled us to insert the 10 amino acids myc-tag at different locations along the α-subunit, in order to better visualize and investigate the DG complex in eukaryotic cells.

**Results:**

We have generated two forms of DG polypeptides *via* the insertion of the myc-tag 1) within a flexible loop (between a.a. 170 and 171) that separates two autonomous subdomains, and 2) within the C-terminal domain in position 500. Their analysis showed that double-tagging (the β-subunit is linked to GFP) does not significantly interfere with the correct processing of the DG precursor (pre-DG) and confirmed that the α-DG N-terminal domain is processed in the cell before α-DG reaches its plasma membrane localization. In addition, myc insertion in position 500, right before the second Ig-like domain of α-DG, proved to be an efficient tool for the detection and pulling-down of glycosylated α-DG molecules targeted at the membrane.

**Conclusions:**

Further characterization of these and other myc-permissive site(s) will represent a valid support for the study of the maturation process of pre-DG and could result in the creation of a new class of intrinsic doubly-fluorescent DG molecules that would allow the monitoring of the two DG subunits, or of pre-DG, in cells without the need of antibodies.

## Background

Dystroglycan (DG) is a widely expressed transmembrane protein that connects the extracellular matrix to the cytoskeleton. It is composed of two subunits, α and β, encoded by a single gene and expressed as a unique precursor (pre-DG) that is cleaved into two proteins by an early post-translational processing [1]. In skeletal muscle DG forms, together with sarcoglycans, sarcospan, syntrophins, and dystrobrevins, the dystrophin-glycoprotein complex (DGC). This complex links the extracellular matrix with the actin cytoskeleton and provides stability to the muscle fiber sarcolemma against contractile forces [1]. DG gene *knockout* in mouse induces premature lethality, indicating that DG plays a crucial role during early embryonic development [2]. Recently, the first mutation associated to a mild form of limb-girdle muscular dystrophy has been identified in the DG gene [3]. However, in several other forms of muscular dystrophies, due to mutations in components of the DGC other than DG, the membrane localization or the glycosylation pattern of α-DG are secondarily perturbed [4]. Furthermore, α-DG can act as a direct receptor for *Arenaviruses* and, in complex with laminin, as a receptor for *Mycobacterium leprae*[5,6].

Inside the cell, β-DG interacts with dystrophin, which binds to the actin cytoskeleton, and may act as a scaffolding platform for signalling and adaptor proteins [7]. The β-DG extracellular domain has been catalogued as a “natively unfolded protein”, characterized by the absence of any well defined three-dimensional structure [8]. The cytoplasmic domain of β-DG has a disordered conformation but harbours a series of conserved sites through which it is capable of binding a plethora of intracellular adaptor proteins [7]. α-DG, a glycosylated peripheral membrane protein associated to β-DG via non-covalent interactions, represents the major link between the cytoskeleton and the extracellular space, since it binds several matrix proteins such as laminin and perlecan [1]. α-DG has a dumbbell-like structure characterized by two globular domains, the N-terminus and C-terminus, separated by a central mucin-like region [9]. The 3D structure of its N-terminal domain consists of two sub-domains that are connected by a flexible region spanning the 161–180 a.a. portion of the molecule [10].

Functional studies on DG expressed in transfected cultured cells are limited mainly by the scarce availability of commercial antibodies. However, specific tags (such as myc, Flag or green fluorescent protein, GFP) terminally fused to the C-terminal end of the β-DG cytodomain have been largely used and greatly helped in the visualisation of the β-DG subunit [11-14]. On the other hand, it is often much more difficult to detect signals relative to α-DG, due to its extensive and heterogeneous glycosylation. In this study, we how the α subunit can be tagged with the myc epitope inserted at specific sites within its N-terminal domain in order to better analyze intracellular and membrane-targeted α-DG and its possible processing within cells.

## Results

### Insertion of a myc probe within the α-DG subunit

**Figure 1:**
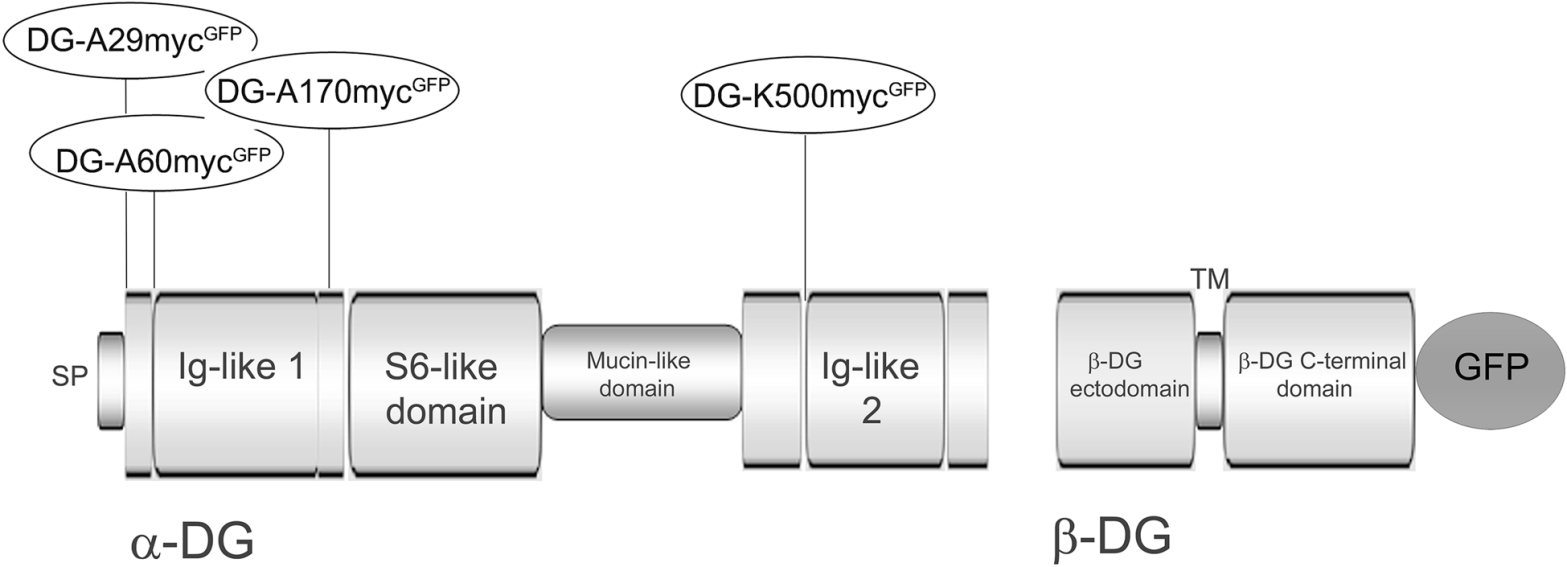
**A schematic cartoon of the domain organization of DG showing the insertion of the myc-tag in different sites within the α-DG subunit.** The myc-tag was inserted at the end of the signal peptide (A29), before the first residue of the solved 3D structure (PDB:1U2C) of the N-terminal region of α-DG (A60), in the middle of the flexible loop connecting the two sub-domains (Ig-like and S6-like) featured within the N-terminal region of α-DG (A170) and right before a second Ig-like domain identified within the α-DG C-terminus (K500). The C-terminal region of β-DG is fused to GFP in the myc-tagged constructs. DG numeral system is based on the majority of mammals sequences available, including the human one, all starting from an additional Met, followed by Arg (1-MRMSV-5), with respect to the murine one (3-MSV-5).

We have used a murine DG cDNA cloned in the pEGFP-N1 vector as a starting template for the insertion of the myc epitope in different positions within α-DG (Figure 1). Specifically, a 10 amino acids myc-epitope (EQKLISEEDL) was intercalated within α-DG in order to visualize by Western blot and fluorescence microscopy the α-DG subunit, that in 293-Ebna cells is very poorly, when not at all, recognized by commercial antibodies.

**Figure 2:**
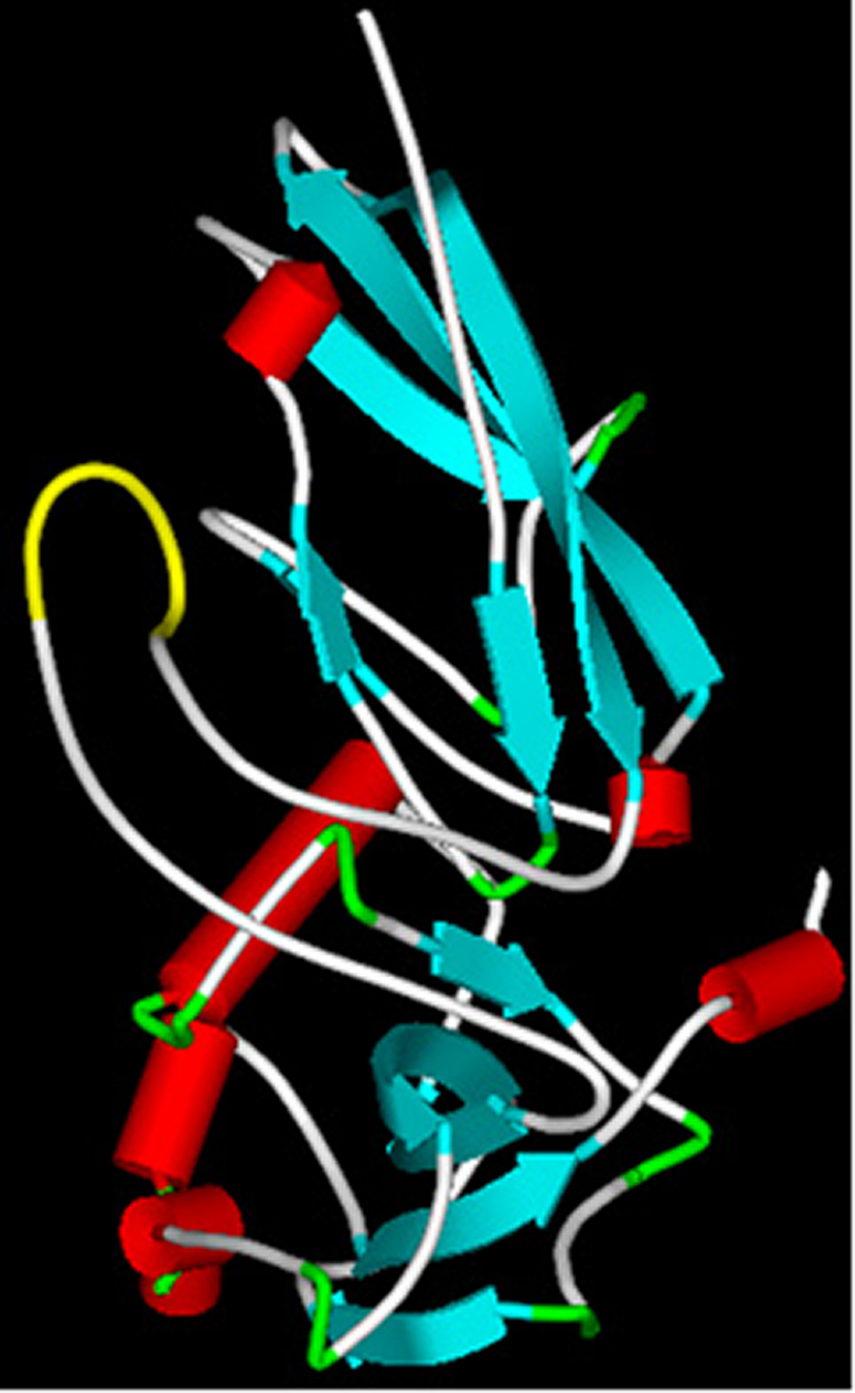
**3D-model of the N-terminus of α-DG with the myc-tag inserted in position 170.** The whole N-terminal region of murine α-DG (positions 3–313 referring to the general numeration based on the human sequence), including i) the ≈ 160-180 endogenous loop region that in our 3D structure could not be solved due to its flexibility [10], and ii) an exogenous 10 a.a. myc-tag inserted between positions 170 and 171, was modeled using the widely used server I-TASSER, which allows *ab initio* modeling of small proteins [16]. The presence of the myc-tag (reported in yellow) does not alter the intrinsic flexibility of this region, which on the contrary remains rather disorganized. Such an insertion is therefore unlikely to influence either the secondary or the tertiary structures of the two surrounding α-DG subdomains, so that the overall folding of the whole α-DG subunit is preserved. Color code for structural elements: light blue (β-strand), red (α-helix), grey (turn or loop) and yellow (myc tag).

We generated different DG constructs characterized by a myc-tagged α-subunit and the C-terminus of the β-subunit fused to the intrinsic fluorescent protein GFP. The insertion of the myc tag was rationally based on the 3D structure of the N-terminal domain of α-DG [10]; we attempted three different positions within such region, namely DG-A29-myc^GFP^, DG-A60-myc^GFP^ and DG-A170-myc^GFP^ (Figure 1). Molecular modeling of the N-terminus of α-DG (based on our available 1U2C PDB structure, [10]) with myc intercalated between positions 170/171, shows that the fold of the α-DG subunit should be totally preserved, as the insertion of the tag does not influence either the local secondary structure or the tertiary structure of the two subdomains (Figure 2). We did not attempt further simulations since our reference (i.e. the recombinant domain-based structure) did not include the α-DG very N-terminal portion (including Ala29, which represents the C-terminal end of the DG signal peptide), and was mostly unstructured at the level of Ala60 [10]. Moreover, on the basis of our recent molecular model of the C-terminal domain of α-DG [15], a myc-tag was also inserted in position K500 (resulting in the construct DG-K500-myc^GFP^), right before the region of the C-terminal domain of α-DG predicted to adopt an immunoglobulin-like fold (see Figure 1).

A technical note that can be of some general interest is that the insertion of 30 nucleotides encoding the myc-tag to produce the DG-A60-myc^GFP^ and DG-A170-myc^GFP^ constructs was readily obtained using the Quick Change mutagenesis kit (Stratagene, USA). All the DNA manipulations and steps taken for intercalating the 30 bp myc-sequences (including the complementary 78 bp primers necessary for producing the two aforementioned constructs) are extensively described in the Materials and Methods section.

### Mature α-DG and myc-tagged α-DG N-terminal domain can be enriched from trasfected 293-Ebna cells

The four constructs were used to transiently transfect 293-Ebna cells, and the resulting exogenous DG molecules were first analyzed by Western blot. It has to be reminded that when total protein extracts are examined, the exogenous β-DG is readily observed: the presence of the EGFP at the C-terminal domain of β-DG increases its molecular mass by about 27 kDa, making the endogenous β-DG (43 kDa) easily distinguishable from the exogenous one (70 kDa).

**Figure 3:**
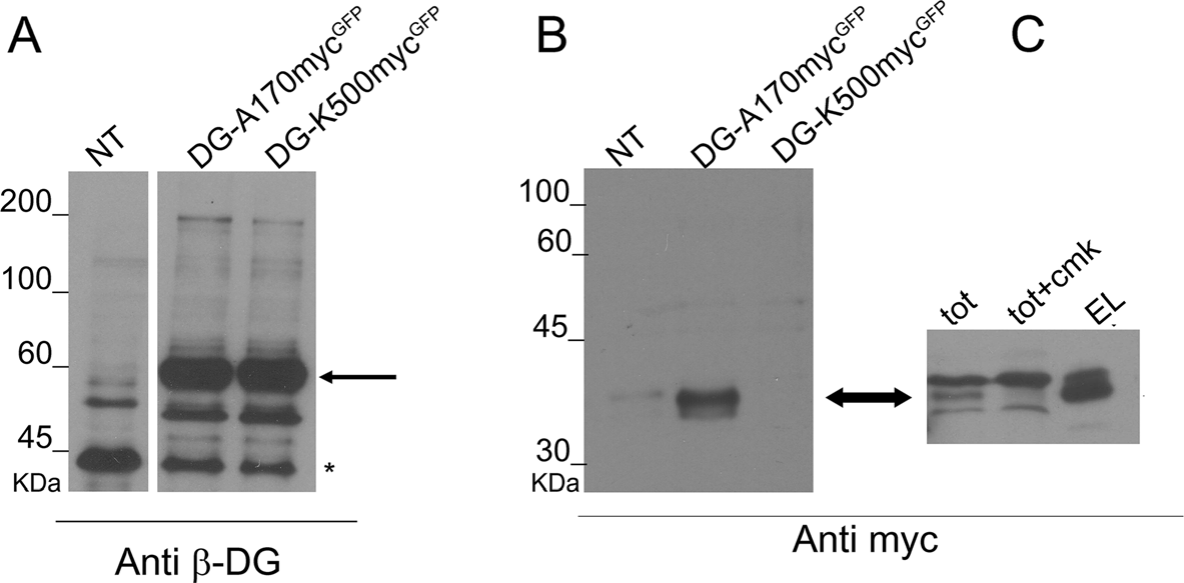
**Western blot of total protein extracts of cells overexpressing DG-A170-myc**^**GFP**^**and DG-K500-myc**^**GFP**^**.** The analysis of total protein extracts was carried out using antibodies directed against β-DG (A) and the myc-tag (B and C). **A**) The insertion of the myc-tag at sites A170 and K500 does not alter the expression and processing of DG, as shown by the presence of a band at ≈ 60 kDa, corresponding to β-DG-GFP (black arrow). Asterisk refers to endogenous β-DG. **B**) Western blot with an anti-myc antibody did not detect any signals in a total protein extract of cells transfected with K500-myc^GFP^, while a band at 34 kDa (black arrow), which is likely to correspond to the α-DG N-terminal fragment harbouring the myc-tag, was present in a total protein extract of cells transfected with A170-myc^GFP^. **C**) Immunoprecipitation with magnetic beads conjugated with an anti-myc antibody of the processed N-terminal domain of α-DG (EL) from a total protein extract of cells transfected with DG-A170-myc^GFP^ (TOT). The N-terminal cleavage is inhibited by CMK added to the cells (TOT + CMK).

Western blot analysis of total protein extracts showed that the insertion of a myc-tag in the construct DG-A29-myc^GFP^ partially inhibited the post-translational processing of the DG precursor, while DG-A60-myc^GFP^ was poorly expressed (data not shown). However, DG-A170-myc^GFP^ and DG-K500-myc^GFP^ were correctly cleaved into α- and β-DG, indicating that the insertion of a myc-tag in these positions does not alter, nor interfere with, the functional maturation of pre-DG (Figure 3A).

Using an anti-myc antibody, we detected a 34 kDa band in the total protein extracts of DG-A170-myc^GFP^ transfected cells that is likely to correspond to the myc-tagged α-DG N-terminal domain processed in the mature protein. In fact, it was proposed that the N-terminal domain of α-DG undergoes an early proteolytic reaction in position 312 mediated by furin, a RER convertase [17,18]. Accordingly, the 34 kDa band is observable in protein total extracts of DG-A170-myc^GFP^ but not in those of DG-K500-myc^GFP^ (Figure 3B). To test whether the myc-tag inserted in the N-terminal domain can be used to specifically isolate and visualise such proteolytic product, we used magnetic beads linked to an anti-myc antibody to immunoprecipitate the α-DG N-terminus from a total protein extract of cells transfected with DG-A170-myc^GFP^. As shown in Figure 3C, a single band of ≈ 34 kDa is purified from the total protein extract, which is likely to correspond to the myc-tagged N-terminal domain. Indeed, the cleavage can be specifically prevented by adding CMK, a furin inhibitor, to the cells (Figure 3C) [18]. This result fully confirms that the proteolytic breakdown at the α-DG N-terminus mediated by furin takes place downstream of position 170 [17,18].

No signals referring to the typical broad pattern of highly glycosylated α-DG were detected in the total protein extracts of DG-K500-myc^GFP^ transfected cells using both anti-αDG (IIH6 and VIA1-4, data not shown) and anti-myc antibodies (Figure 3B). This data suggests that in the total protein extract the level of myc-tagged α-DG is below the range detectable by antibodies, as it is often observed with endogenous α-DG when using commercially available antibodies (data not shown).

**Figure 4:**
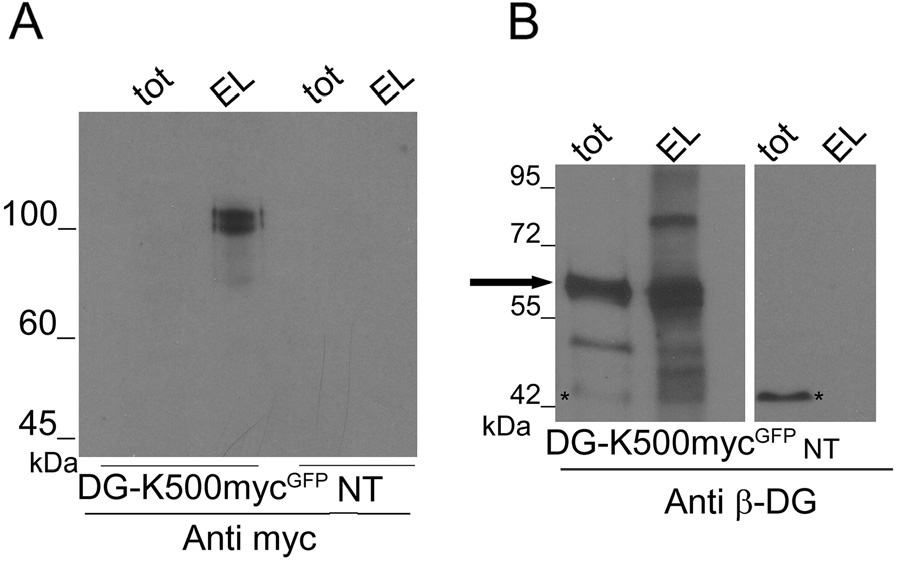
**Enrichment of the myc-tagged α-DG from total protein extracts.** Immunoprecipitation of the myc-tagged α-DG (EL) with magnetic beads conjugated with an anti-myc antibody from a total protein extract of cells transfected with DG-K500-myc^GFP^ (TOT). **A**) Western blot was performed using an anti-myc-HRP conjugated antibody, which detects a band at 120 kDa. TOT, total protein extracts; NT, not transfected cells. **B**) Western blot performed using an anti β-DG antibody showing that α-DG-K500-myc still binds to β-DG since β-DG-GFP is found within the anti-myc pulled-down fraction. In cells transfected with DG-K500-myc^GFP^ , the band at 60 kDa corresponds to β-DG-GFP (black arrow), while in not transfected cells the band at 43 kDa (asterisk) corresponds to endogenous β-DG.

However, when α-DG was enriched by immunoprecipitation with an anti-myc antibody conjugated to magnetic beads, a 120 kDa band representing the fully glycosylated α-DG subunit expressed in embryonic kidney cells [19] was clearly revealed by Western blotting using an anti-myc-HRP-conjugated antibody (Figure 4A). Such immunoprecipitated α-DG was not reactive to IIH6 and VIA1-4 antibodies (data not shown), indicating that in Ebna-293 cells α-DG is not recognized by these commercial antibodies, as already shown by other works [11,20].

Other interesting aspects of our K500-myc tagged α-DG, pointing to its full functionality, are i) that it can still bind to β-DG, as the β-subunit can be enriched in immunoprecipitation with an anti-myc antibody (Figure 4B) and ii) it is still able to pull-down ERp57, a novel potential DG interactor [21]. Such evidences confirm that α-DG-K500-myc shares similar structural and functional properties with wild-type α-DG. Moreover, it is noteworthy that also in a different cell type, likely characterized by a different glycosylation pattern of α-DG, the DG-K500-myc^GFP^ construct encodes for two DG subunits that appear to be highly expressed and correctly targeted to the plasma membrane (LNCaP prostate cancer cells, Steve Winder, personal communication).

### Confocal microscopy of myc-tagged DG-GFP constructs

**Figure 5:**
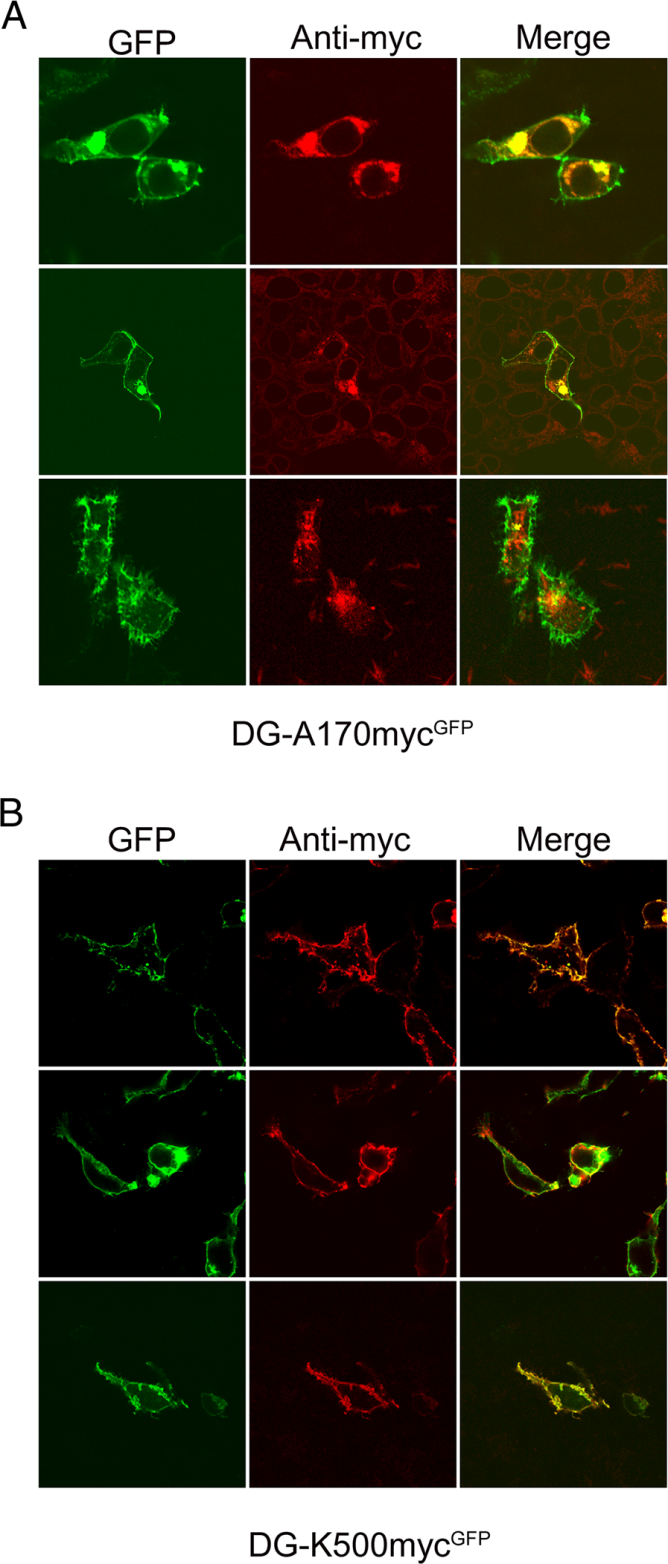
**Confocal microscopy of myc-tagged DG constructs expressed in 293-Ebna cells.** GFP and c-myc immunofluorescence staining of cells transfected with DG-A170-myc^GFP^**A**), and DG-K500-myc^GFP^**B**), constructs. GFP specific signal at 488 nm excitation, c-myc immunostaining at 543 nm excitation and their merge are shown. A XY confocal section is represented for each signal. **A**) and **B**) The GFP signal (green) shows that from both the DG-A170-myc^GFP^ and DG-K500-myc^GFP^ constructs originate a β-DG-GFP protein that localizes at the plasma membrane in a way reminiscent of the wild-type β-DG-GFP. **A**) C-myc immunostaining shows no plasma membrane signal in cells transfected with the DG-A170-myc^GFP^ construct, but only intense diffused and punctuated perinuclear (red) signals. **B**) The DG-K500-myc^GFP^ construct instead shows a strong plasma membrane localized c-myc immunostaining (red), whose localization is fully confirmed by the yellow-orange colour observable in the merged images.

Confocal microscopy of fixed cells transfected with the myc-tagged constructs was used to analyze the subcellular localization of the DG complex (Figure 5). Both the myc-tagged constructs were able to originate a β-DG-GFP product that was exported to the plasma membrane as suggested by the analysis of the GFP-intrinsic fluorescence signals in Figure 5A (DG-A170-myc^GFP^) and Figure 5B (DG-K500-myc^GFP^) that are similar to the wild-type β-DG-GFP (data not shown, see [20]).

There is nearly no detectable c-myc immunostaining signal at the plasma membrane for DG-A170-myc^GFP^, for which instead more signal is observed around the nucleus, presumably at the ER and Golgi compartments (Figure 5A). As mentioned above, this experimental evidence strongly points toward the full shedding of the N-terminal portion of α-DG [17]. On the contrary, DG-K500-myc^GFP^ displays a clear plasma membrane staining when cells are stained with the anti-c-myc antibody, suggesting that tagged α-DG is correctly targeted to the plasma membrane (Figure 5B).

## Discussion

### Myc-tagging the α-DG domains: finding the best structurally permissive site(s) for an improved biochemical and microscopic detection

Short linear epitope tags that confer specific targeting properties, including an enhanced affinity for interactors (proteins, enzymes, antibodies), or the possibility for a facilitated detection by fluorescence microscopy, are extensively used as fundamental tools in cellular biochemistry analyses.

Like other investigators, we have fused to the C-terminal end of the β-DG cytoplasmic domain the green fluorescence protein (GFP), which allowed to better identify the β-subunit of DG [20]. The data collected so far show that the presence of the chimaeric 27 kDa GFP subunit does not compromise or influence the adhesion and signalling function of β-DG anyhow, implying that no major steric hindrance effects can be ascribed to the intrinsically fluorescent partner. On the other hand, the α-subunit of DG is much less easy to detect, due to its extensive glycosylation shell that can prevent the binding of antibodies, depending on the high negative charge of the syalic terminations or simply on their hindrance. In fact, only few commercial antibodies that recognise undisclosed carbohydrate epitopes protruding from the α-DG subunit are currently available, and sometimes they generate faint signals, if at all, especially on some transfected cell lines. Such behaviour could depend on the fact that every cell line has its own repertoire of glycosyltransferases, so that exogenous DG molecules could be differently decorated with sugars depending on the cell line employed.

In this paper we have focussed our attention on α-DG produced in the commonly used 293-Ebna cell line. The knowledge that we have accumulated on the domain organization of α-DG was fundamental in the rational search of a permissive site to be used for the insertion of a specific tag (a 10 amino acids myc-epitope) for better detecting and visualising the α-subunit of DG in transfected cells without disrupting the structural organization of the DG-complex.

The insertion of the myc-tag was rationally designed based on the three-dimensional structure of the N-terminal domain of α-DG [10] (Figure 2) and on the model of its C-terminal domain [15]. The X-ray structure of the N-terminal domain, spanning the amino acids 30–315, showed the presence of two sub-domains: the first identified as an Ig-like domain, between amino acids 60–160, and the second resembling the ribosomal RNA-binding protein S6, between amino acids 181–315, with a very flexible region (161–180) interconnecting the two sub-domains. The tag was then inserted at the end of the signal peptide of pre-DG (DG-A29-myc^GFP^) [22], and before and after the Ig-like domain (DG-A60-myc^GFP^ and DG-A170-myc^GFP^). On the basis of a model of the C-terminal domain of α-DG, the myc tag was also inserted in position K500, just in front of a region predicted to fold as an Ig-like domain (DG-K500-myc^GFP^).

Western blot analysis of total protein extracts of transfected cells showed that the presence of the myc-tag at the positions A170 and K500 did not affect the expression levels of DG and the processing of the DG precursor into the two subunits (Figure 3). Notably, the insertion of the myc tag at the C-terminal domain of α-DG (DG-K500-myc^GFP^) allowed to visualize the α-DG subunit in Western blot after immunoprecipitation and in fluorescence microscopy (Figures 4A and 5B, respectively). This result is particularly interesting due to the typical difficulties in recognizing α-DG with the plethora of commercial antibodies directed towards some of its undefined carbohydrate epitopes. Moreover, the insertion of 10 amino acids does not perturb the interaction between α- and β-DG, and the myc-tagged α-DG is still able to pull-down ERp57 [21], suggesting that the tagged α-DG is functionally similar to wild-type α-DG [20].

Besides, the insertion of myc-tag can be used successfully to enrich the N-terminal domain of α-DG from cellular extracts. Recently, it was proposed that the N-terminal domain of α-DG would serve as a recognition and anchoring site for the glycosyltransferase LARGE and, upon glycosylation, would be then cleaved in the mature protein [17,18]. The corresponding fragment was found in the medium of different cell types and circulating in the human serum, although in very limited amounts [23].

In our Western blot experiments carried out on total protein extracts derived from cells transfected with the DG-myc tagged constructs, a band running at ≈ 34 kDa is observable in DG-A170-myc^GFP^ but not in DG-K500-myc^GFP^ (see arrow in Figure 3B and C). In total cellular extracts this band is likely to represent some cleaved myc-tagged N-terminal portion of α-DG that engulfs the cytosol/ER and/or is ready to be delivered outside the cells via some exocytosis-based pathway. Our initial attempts to collect the band from the medium culture failed, due to the very low amount of protein available (data not shown). However, exploiting anti-myc antibodies immobilised on magnetic beads, we showed that the myc-tag inserted in position A170 can be used to specifically collect increased amounts of the processed N-terminal domain of α-DG. In fact, a single band of 34 kDa can be isolated from the total protein extract of transfected cells and we will use it for further biochemical characterization, including mass spectrometry (Figure 3B and C). Indeed, in a recent paper reporting the characterization of recombinant murine α-DG, in the absence of an appropriate N-terminal tag the Authors could not find any peptides derived from the entire N-terminal portion of α-DG [24]. All considered, the rational design of myc-intercalated α-DG constructs that we have carried out based on our extensive expertise on domain organization and structural aspects of α-DG [10,15] proved to be a very powerful strategy for improving the detection of α-DG molecules, and their possible fragments, within cells or in cellular extracts.

## Conclusions: towards an intrinsic doubly fluorescent dystroglycan?

In the future novel chimaeric constructs will be also tested in additional cell lines, in order to further validate their properties and to check whether any differences within the glycosylation or maturation pathway of DG would influence their stability.

Some of the identified insertion sites (or even additional ones) still represent good candidates for further molecular manipulations that ambitiously aim at obtaining a DG carrying two different intrinsic fluorophores, thus creating a doubly-fluorescent molecule [25,26]. Such a strategy will make it possible to analyze the behavior of DG in living cells without all the potential artefacts that can arise from antibody staining.

A doubly fluorophore-tagged DG could be very useful in the investigation, by confocal microscopy technologies, of the dynamic details of the pre-DG maturation and N-terminal α-DG processing in living cells (along the ER-Golgi-Plasma Membrane *route*), and in any case could be of help in studies aimed at determining the targeting of the two DG subunits (or of pre-DG) at the plasma membrane or at other subcellular compartments. In fact, measuring the fluorescence resonance energy transfer (FRET) between the two fluorophore-tagged DG subunits would enable to monitor interactions of these proteins in living cells over time, and under different experimental conditions.

## Methods

### DNA manipulations

The full-length cDNA encoding for murine DG was used as a template to generate by the *gene splicing by overlap extension* technique (GENE SOEing) [27] two overlapping DNA constructs which allowed, by a third PCR reaction (see below), to insert the c-myc epitope upon the triplet encoding for K500.

The following primers were used to generate DG-K500-myc^GFP^ :

PCR A:

forward primer

5'-ccc**gaattc**atgtctgtggacaactggctactg-3′

reverse primer

5′-**cagatcatcctcttctgagatgagtttttgttc**cttgagctctggccgctggttaggttctcc −3′

PCR B:

forward primer

5′- **gaacaaaaactcatctcagaagaggatctg**aatcacattgacagggtagatgcctgggtg −3′

reverse primer

5′- ccc**gaattc**ttaagggggaacatacggaggggg-3′

The EcoRI restriction sites and the c-myc epitope sequence are given in bold type. The two GENE SOEing final products were obtained by a third PCR reaction using the two purified PCR A and PCR B and the following primers:

forward primer

5′-ccc**gaattc**atgtctgtggacaactggctactg

reverse primer

5′- ccc**gaattc**ttaagggggaacatacggaggggg-3′

The PCR products were subsequently cloned in the pEGFP-N1 vector (Clontech, USA).

The DG-A170-myc^GFP^ were directly produced using the QuikChange site-directed mutagenesis kit (Stratagene, USA) with the following primers (the c-myc epitope sequence is in bold type), using pEGFP-N1-DG as a template :

DG-A170-myc^GFP^

forward primer

5′-gagccacagtctgtacgggcagcc**gaacaaaaactcatctcggaagaggatctg**tcatcagaccctggtgaggtagtg

reverse primer

5′-cactacctcaccagggtctgatga**cagatcctcttccgagatgagtttttgttc**ggctgcccgtacagactgtggctc

All the final constructs were verified by automated sequencing.

### Cell culture and transfection

293-Ebna cells were grown in DMEM supplemented with antibiotics and 10 % (v/v) fetal calf serum. About 20 μg of vector containing the constructs were used to transfect 293-Ebna cells using the calcium phosphate method. Briefly, DNA was mixed with 125 mM CaCl_2_ and BES-buffered saline, containing 50 mM BES, 280 mM NaCl and 150 mM Na_2_HPO_4_. The DNA calcium phosphate complex was added to the cells. After 24 h, cells were collected for Western blot analysis. For the inhibition of furin, 20 μM CMK was added to the cells for 24 h after transfection. For fluorescence and confocal microscopy analyses, 5 μg of each construct were transiently transfected into 293-Ebna cells, and after 24 hours cells were fixed with 4 % (v/v) paraformaldehyde at room temperature for 10 min.

### Total protein extraction and Western blot

Cells transfected with the four constructs, with empty pEGFP and wild-type DG-GFP were lysed with PBS containing 1 % Triton X-100 and protease inhibitors (Roche, Switzerland), then centrifuged at 14000 rpm for 10 min at 4 °C. After 1 h of incubation at 4 °C, 20 μg of each lysate was resolved on a 10 % SDS-PAGE. For Western blot analysis, proteins were transferred to nitrocellulose and probed with the following antibodies diluted in the PBS containing 0.05 % Tween 20 and 3 % BSA: anti β-DG (Novocastra, UK) (1:50) and anti c-myc-HRP conjugated (Miltenyi Biotec., Germany) (1:5000). The nitrocellulose was incubated with peroxidase-conjugated secondary antibody (Sigma, USA) diluted 1:7000 (anti-mouse); the reactive products were revealed using the luminol-based ECL system (Pierce, USA).

### Immunoprecipitation

All the steps required for immunoprecipitation were carried out using the µMACS Epitope Tag Protein Isolation Kit (Miltenyi Biotec., Germany) , following the manufacturer’s instructions. Briefly, 1 ml of total protein extract of transfected cells was incubated with 50 μl of magnetic beads conjugated with an anti-myc antibody (Miltenyi Biotec., Germany) for 30 min at room temperature. After several washes, the adsorbed protein was eluted with 50 μl of sample buffer, incubated at 99^○^C and run on a 10 % SDS-PAGE followed by Western blot analysis with an anti-myc antibody-HRP conjugated (Miltenyi Biotec., Germany).

### Confocal microscopy

Transiently transfected 293-Ebna cells were fixed and permeabilized in PBS containing 0.1 % Triton X-100 at room temperature for 10 min followed by incubation with the anti c-myc antibody (Sigma, USA) diluted 1:1000 in PBS containing 0.1 % Triton X-100. After several washes the cells were incubated with a secondary antibody conjugated to rhodamine (Invitrogen, USA) (1:500), imaged with a confocal laser scanning system (TCS-SP2, Leica Microsystems, GmbH, Wetzlar, Germany) and analysed with ImageJ programs (http://rsbweb.nih.gov/ij/). Laser excitation at 488 nm of the sample was followed by an excitation at 543 nm to collect emission signals from GFP and rhodamine conjugated antibodies, respectively.

## Abbreviations

DG, Dystroglycan; DGC, Dystrophin-glycoprotein complex; GFP, Green fluorescent protein; CMK, Furin inhibitor I decanoyl-Arg-Val-Lys-Arg-chloromethylketone; GENE SOEing, Gene splicing by overlap extension; PDB, Protein data bank; FRET, Fluorescence resonance energy transfer.

## Competing interests

The authors declare that they have no competing interests.

## Authors’ contributions

SM prepared the initial panel of constructs and carried out Western blotting and immunoprecipitation; FS carried out Western blotting, immunoprecipitation and confocal microscopy and participated in writing the manuscript; MGB contributed to the preparation of the K500 myc-tag construct and in the critical revision of the manuscript; MB contributed to results analysis; WH carried out confocal microscopy experiments; AG revised the manuscript; BG revised the manuscript; AB conceived and directed the project, contributed to the experimental design of the study and wrote the manuscript. All authors read and approved the final manuscript.
